# Cost-Effectiveness of Anticoagulation in Patients with Nonvalvular Atrial Fibrillation with Edoxaban Compared to Warfarin in Germany

**DOI:** 10.1155/2015/876923

**Published:** 2015-03-17

**Authors:** Martin Krejczy, Job Harenberg, Martin Wehling, Konrad Obermann, Gregory Y. H. Lip

**Affiliations:** ^1^Department of Clinical Pharmacology, Medical Faculty Mannheim, Ruprecht-Karls University Heidelberg, Maybachstraße 14, 68169 Mannheim, Germany; ^2^Mannheim Institute of Public Health, Medical Faculty Mannheim, Ludolf-Krehl Straße, 68167 Mannheim, Germany; ^3^Centre for Cardiovascular Sciences, City Hospital, University of Birmingham, Birmingham B187QH, UK

## Abstract

We compared the cost-utility analysis for edoxaban at both doses with that of dabigatran at both doses, rivaroxaban, and apixaban (non vitamin K antagonist oral anticoagulants, NOAC) in a German population. Data of clinical outcome events were taken from edoxaban's ENGAGE-AF, dabigatran's RE-LY, rivaroxaban's ROCKET, and apixaban's ARISTOTLE trials. The base-case analyses of a 65-year-old person with a CHADS2 score >1 gained 0.17 and 0.21 quality-adjusted life years over warfarin for 30 mg od and 60 mg od edoxaban, respectively. The incremental cost-effectiveness ratio was 50.000 and 68.000 euro per quality-adjusted life years for the higher and lower dose of edoxaban (Monte Carlo simulation). These findings were also similar to those for apixaban and more cost-effective than the other NOAC regimens. The current market costs for direct oral anticoagulants are high in relation to the quality of life gained from a German public health care insurance perspective. The willingness-to-pay threshold was lowest for 60 mg edoxaban compared to all direct oral anticoagulants and for 30 mg edoxaban compared to dabigatran and rivaroxaban.

## 1. Introduction

In a large prospective randomized double blind and double dummy study (ENGAGE-AF), once-daily dosing of the oral direct factor Xa inhibitor edoxaban 60 mg (dose adjusted to 30 mg) was noninferior warfarin for the prevention of the primary endpoint of stroke and systemic embolic events in patients with nonvalvular atrial fibrillation (NVAF), when compared to warfarin with INR (international normalized ratio) adjusted to 2 to 3; edoxaban 60 mg was also associated with a significantly lower rate of major bleeding and cardiovascular mortality [1]. Other nonvitamin K oral antagonist oral anticoagulants (NOAC, dabigatran, rivaroxaban, and apixaban) have proven to be superior or at least equivalent for stroke prevention and occurrence of severe bleeding complications in patients with NVAF compared to dose-adjusted warfarin. In the RE-LY trial 110 mg bid dabigatran was noninferior and 150 mg bid dabigatran was superior to dose-adjusted warfarin for prevention of stroke and systemic embolism and both doses resulted in less intracranial bleeding [2]. In the double blind ROCKET-AF trial patients on rivaroxaban 20 mg od had reduced rates of stroke and systemic embolism and comparable major bleeding incidences compared to warfarin [3]. In the double blind ARISTOTLE trial, apixaban was associated with lower rates of strokes and major bleeding and reduced incidence of cardiovascular deaths compared to warfarin [4].

One of the main limitations for prescribing NOAC in real life is their higher daily price compared to warfarin. The pharmacoeconomic aspects of dabigatran, rivaroxaban, and apixaban were analysed for many countries. All have been demonstrated to be cost-effective in many countries for the health care system mainly based on the reduced incidence of major bleeding complications but also for some of the NOAC, due to a lower incidence of ischemic stroke and systemic embolism [5]. Related analyses include a willingness to pay, an incremental cost-effectiveness ratio (ICER) of a currency (euro, USD, or any other currency) per quality-adjusted life years (QALY) for dabigatran, rivaroxaban, or apixaban compared with warfarin. As the results of all these investigations were similar regarding the socioeconomic benefit of NOAC compared to warfarin, it was argued if these analyses have to be performed separately for every country. National guidelines for economic evaluation agree that a given country's unit costs should be applied to calculations of costs when adapting an analysis for local decision making [6]. As an example, lower prices for dabigatran in Europe compared with those in the USA were resulted in more favourable cost-effectiveness ratios despite smaller estimated gains in quality-adjusted survival [7]. Here we determined the cost-effectiveness of 60 mg od and 30 mg od edoxaban from a German payers perspective and compared the results with those obtained for the approved NOAC dabigatran (both doses 110 mg bid and 150 mg bid), rivaroxaban, and apixaban [5].

## 2. Methods

### 2.1. Markov Decision Model and Data Sources

**Figure 1:**
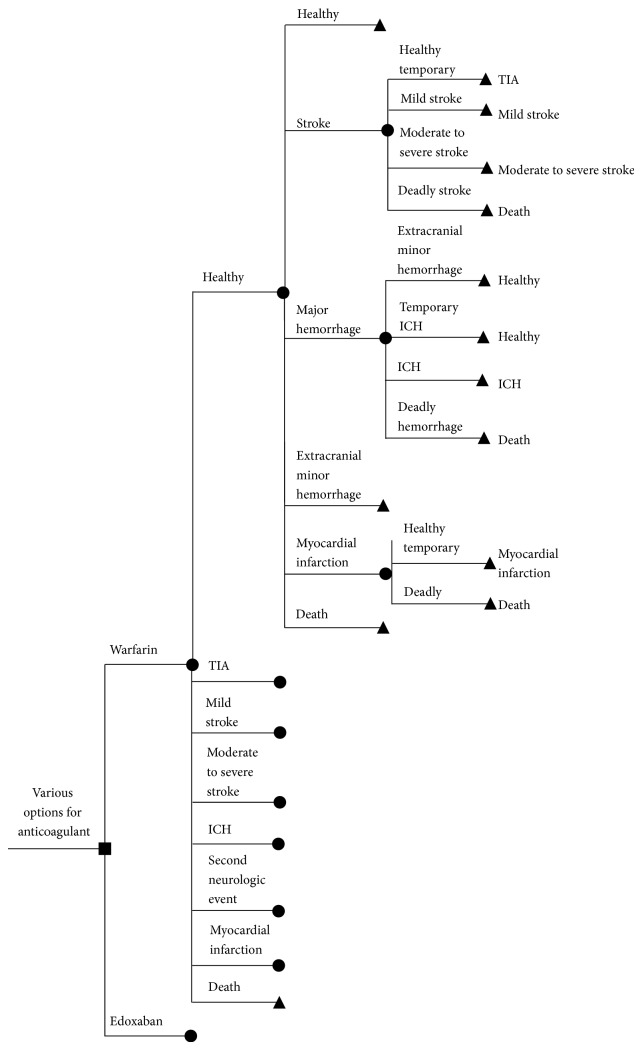
Outline of the Markov model for data of the ENGAGE-AF study. Here one dose of edoxaban is given as an example used in the Markov model. ICH intracerebral haemorrhage; TIA transient ischemic attack (modified from [5]).

We used the Markov decision model to analyse the QALYs, total costs (one-time costs for events, rehabilitation costs for inpatient and ambulatory care, inpatient medical treatment costs, and daily costs for drugs), and ICER based on the data of the ENGAGE-AF [1] study. The results were compared with our data previously derived from the RE-LY, ROCKET-AF, and ARISTOTLE trials [5] under a German health care insurance perspective. The following health states and outcome events were included: healthy with NVAF, transient ischemic attack, ischemic stroke (fatal, moderate to severe, and mild), haemorrhage (fatal, moderate to severe intracranial, mild intracranial, major noncerebral, and minor noncerebral), myocardial infarction (MI), recurrent and combined events, and cardiovascular mortality using the results from the ENGAGE-AF trial and costs for the German population [8] (Figure 1). Definitions of these events were taken from the ENGAGE-AF study [1]. Event probabilities were not included if they were not reported consistently across the studies (systemic embolism, pulmonary embolism, hemorrhagic stroke, and bleeding in other locations) (Table 1).

For the base-case analysis we used a hypothetical population cohort of patients with the starting age of 65 years with NVAF who were at increased risk for stroke (CHADS2-score >1) with no contraindications to anticoagulation as reported in the ENGAGE study [1]. Our results were expressed in quality-adjusted life years (QALY), 2012 euro, and incremental cost-effectiveness ratios (ICER: total costs (€, edoxaban) - total costs (€, warfarin)/QALY (edoxban) - QALY (warfarin)). We applied utilities and costs to each outcome yearly or event driven and discounted costs and benefits at 5% annually [9, 10]. A half cycle correction was done for each model, using a cycle length of 1 year. We quantified QALYs, risk for adverse events, and net cost for a time horizon of 20 years for the German population [11]. This time frame is used for CEA investigations taking well in account the much shorter treatment period of the studies from which the data are used (12).

### 2.2. Probability of Adverse Outcome Events and of Endpoints

The adverse outcome events and endpoints with the 95% confidence intervals (CIs) were taken from the ENGAGE-AF study [1] and were found to be similar with those reported by Freeman et al. [12]. Intention to treat (ITT) values were taken for ischemic stroke, myocardial infarction, death from cardiovascular cause, and death from nonvascular cause and on-treatment values (OT) for bleeding events (minor bleeding, major bleeding, and ICH) for calculations in the sensitivity analyses (Table 1).

### 2.3. Severity of Stroke and Haemorrhage

Ischemic stroke was classified into fatal, moderate to severe, mild, and no neurologic deficit (4 categories) as reported [12]. A second mild ischemic stroke was defined to result in a moderate to severe ischemic stroke or death and a second moderate stroke in a severe ischemic stroke, reduced life quality, or death [13].

Haemorrhage was categorized into fatal, ICH with moderate to severe neurologic sequelae, ICH with no neurological deficit, major extracerebral haemorrhage, and minor extracerebral haemorrhage [12, 13]. A moderate to severe ischemic stroke followed by an ICH was categorized into a moderate to severe neurologic outcome. Decrease of quality of life depended on the severity of outcome and resulted in different costs according to the German health care system [14].

### 2.4. Mortality Rates

The mortality rates (death from vascular cause and death from any other cause) were taken from the ENGAGE-AF study [1]. The annual rates for death from any other cause were taken from published German mortality tables [8].

### 2.5. Quality of Life Utilities

The quality-adjusted life years (QALY = survived life years adjusted for quality of life) [15] were calculated by multiplying the time spent within a health state with the corresponding utility value. The utility values for warfarin were taken from data on patients with NVAF who underwent time trade-off and standard gamble methods to estimate their quality of life [12, 16]. All utility values were discounted in our model [17]. A utility of “1” represents a completely healthy status and a utility of “0” represents death. The mean utility for warfarin was 0.987 [12, 16, 18]. The utility for edoxaban was estimated as published for ximelagatran [12, 18], dabigatran, rivaroxaban, and apixaban [5, 12, 13, 16, 18–22].

### 2.6. Costs for Drugs and Outcome Events

One-time costs for most events were taken from the institute for payment regulations in German hospitals (Institut für Entgeltsystem im Krankenhaus,* InEK*) which included German-diagnosis related groups (G-DRGs) [14] and were expressed in euro and reflected from the health care insurance perspective in Germany in 2012. Costs for bleeding events are not included in the G-DRGs and were taken from the literature [23]. Of note, our analysis did not include indirect costs and they were calculated over a time horizon of 20 years for the German population [11] with a discount of 5% (0% and 10% in the sensitivity analyses) per year [9, 10, 24, 25]. Rehabilitation costs were included following ischemic strokes, ICH, and myocardial infraction, but not major bleedings without need for rehabilitation.

Cost for warfarin therapy, a mean of three-week interval for INR measurements with patient office visits, was set at 153€ annually [26, 27]. The retail costs and daily costs for edoxaban according to costs of other NOACs (3.37 Euro per day) and phenprocoumon (0.20€ per day) were taken from pharmacies and the “red list” (German equivalent of “The Physicians' Desk Reference Manual” in USA, e.g.) [26]. Total costs for the drugs warfarin respected the event costs according to the* InEK*. These entries were used to examine the cost-effectiveness based on the event probabilities (Tables 1 and 3).

### 2.7. Sensitivity Analyses

One-way sensitivity analyses of all variables were included in the decision models over their plausible ranges. Ranges for clinical events were derived from confidence intervals (CI) for event probabilities [1]. Medication costs for edoxaban and phenprocoumon were included as reported above. Two-way sensitivity analyses were performed for combinations of stroke and ICH using the values of warfarin [1].

### 2.8. Probabilistic Sensitivity Analysis

The Monte Carlo simulations (MCS) were made using random sampling and random distribution of variables for 10 000. Beta-distribution of the event probabilities was assumed for the calculation except for subcategories of stroke using Dirichlet distribution [12]. The Dirichlet distribution was chosen to show the probability of our subclassifications. Maximum and minimum ranges of costs for each adverse event were calculated using the German* InEK* and a gamma- and log normal distribution.

### 2.9. Statistical Methods

The models and analyses were created with TreeAge Pro 2013 and Microsoft Excel 2003.

## 3. Results

### 3.1. Base-Case Analysis, One-Way Sensitivity Analyses, and Two-Way Sensitivity Analysis

The calculated QALY, the total costs, and the ICER with edoxaban and warfarin are shown in Tables 2 and 3. For comparison the data derived from the outcome events of the RE-LY, ROCKET-AF, and ARISTOTLE trials are included as published [5]. The ICER for edoxaban 60 mg was lower compared to edoxaban 30 mg daily. Compared to the other NOAC regimens, edoxaban 60 mg had the lowest ICER (Table 4). This difference is mainly due to the lower QALY of the warfarin group in the ENGAGE-AF study compared to the other studies.

The results of the one-way sensitivity analysis showed that the costs for edoxaban (both doses), the quality of life utilities, the treatment of ischemic stroke, and the treatment of major and intracerebral bleeding complications were important values in our model. Edoxaban and apixaban thus were most cost-effective compared to the other NOAC [5].

### 3.2. Two-Way Sensitivity Analyses

The two-way sensitivity analyses of key variables for varying risk rates for ischemic stroke and intracerebral haemorrhage showed that both doses of edoxaban were preferable as a therapy for combinations of moderate to high risks for ischemic stroke and high risk of intracerebral haemorrhage at a set willingness to pay of 50.000€ per QALY against INR-dose-adjusted warfarin. These findings were also similar to those for apixaban and more cost-effective than the other NOAC regimens [5]. Using the probabilistic sensitivity analysis (PSA) in the Monte Carlo simulation by varying all variables simultaneously resulted in a willingness-to-pay threshold (PSA ICER) of 52.000€ per QALY for edoxaban 60 mg od and 69.600€ per QALY for edoxaban 30 mg od (Table 4). A similar market price was assumed for edoxaban at both doses as for the other NOAC. These data were similar to the base-case results and similar to apixaban and lower than for rivaroxaban and dabigatran at both doses as reported earlier [5]. The analysis demonstrates the cost-effectiveness of both doses of edoxaban for prevention of cerebral and noncerebral embolic events in patients with nonvalvular AF. The ICERs are comparable to apixaban and lower compared to dabigatran at both doses and rivaroxaban, despite an almost identical daily price for all NOACs.

### 3.3. Probabilistic Sensitivity Analyses: Monte Carlo Simulation

**Figure 2:**
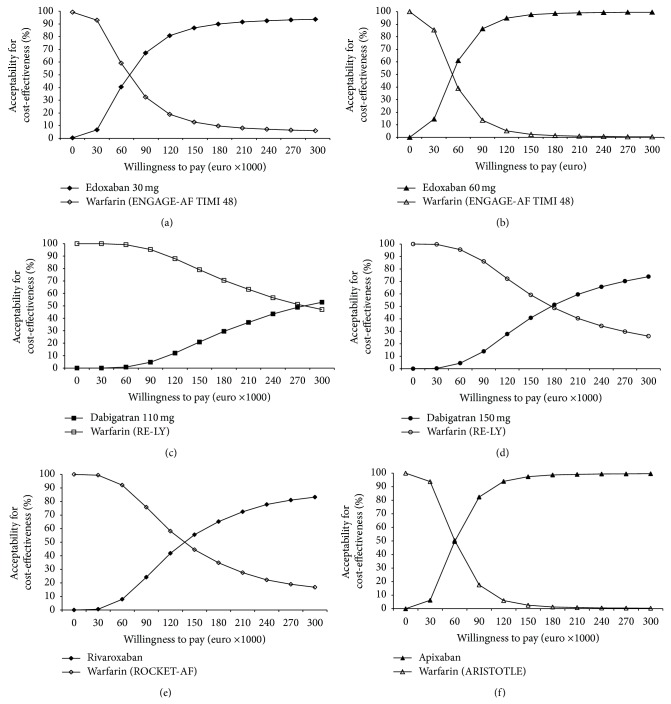
Monte Carlo simulation: acceptability curve for edoxaban 30 mg od (a), edoxaban 60 mg od (b), dabigatran 110 mg bid (c), dabigatran 150 mg bid (d), rivaroxaban 20 mg od (e), and apixaban 5 mg bid (f) compared to warfarin (results obtained from data of every NOAC study) with current market prices for a population starting with 65 years from a German health insurance perspective (reproduction of (c) to (f) with permission of the publisher of [5]).

The various willingness-to-pay thresholds were analysed by using the probabilistic sensitivity analysis (PSA) in the Monte Carlo simulation (MCS) and varying all variables simultaneously. As a result edoxaban 60 mg od and edoxaban 30 mg od were cost-effective at willingness-to-pay threshold of 52.000€ per QALY and 67.000€ per QALY higher (Figure 2). The results of the cost-effectiveness at willingness-to-pay thresholds for the other NOAC treatment regimens are shown for comparison at Krejczy et al. [5]. The PSA results were similar to the base-case results.

### 3.4. Subgroup Analyses

Total costs increased and the ICER decreased in a base-case analysis for a 65- to 85-year-old cohort from the German public health care insurance perspective excluding a discount for costs and utility values.

The absolute numbers of QALY and total costs decreased when costs and utility values were discounted by 10%. In the same time the ICER increased in a base-case data for a 65- to 85-year-old cohort from the German public health care insurance perspective.

## 4. Discussion

The present study shows that the two dosage regimens of edoxaban 60 mg od and edoxaban 30 mg od are nearly cost-effective bid for prevention of ischemic stroke and systemic embolic events in patients with NVAF based on the data of the ENGAGE-AF study based on a societal willingness to pay comparable to data from other countries. Comparing these data obtained with the other four treatment regimens with dabigatran 110 mg bid, dabigatran 150 mg bid (RE-LY study [2]), rivaroxaban 20 mg od (ROCKET-AF study [3]), and apixaban 5 mg (ARISTOTLE trial [4]) which are available in Germany and many other countries edoxaban 60 mg was the most cost-effective followed by apixaban 5 mg bid, edoxaban 30 mg od, and the 3 remaining treatment regimens (Table 4). Of note these data were obtained using the same inputs into the Markov model based on the German insurance system. From the public health care insurance view, only edoxaban 60 mg od treatment was nearly cost-effective at a hypothetical willingness-to-pay threshold of 50.000 EUR for patients at a moderate or higher risk of stroke (CHADS2-score >1) compared to INR-adjusted warfarin with current German market costs.

Similar analyses were reported for the two doses of dabigatran based on the health care costs and willingness to pay in USA [12, 13], Canada [28], United Kingdom [29, 30], Denmark [31], Sweden [32], and Portugal [33], for rivaroxaban in USA [34], for all three NOACs in USA [35], Canada [36], Germany [5], and Italy [37], and as a comparative analysis for dabigatran and rivaroxaban in Canada [38]. All analyses for dabigatran used the Markov model for calculation of the QALYs and ICERs and a one-way and two-way sensitivity analysis. In addition, we calculated these data for rivaroxaban and apixaban as well as for a certain range of daily costs for warfarin and daily costs of the NOACs for Germany. The cost data we used for the Markov model were comparable to those used in other countries [12, 28–31, 34].

Despite differences in model designs and structures of the cost-effectiveness analyses, it was mostly possible to replicate the results published by different authors in different countries like USA, UK, and Canada and identify variables responsible for differences between ICERs using a reference model approach [39]. All analyses for dabigatran used the Markov model for calculation of the QALYs and ICERs and a one-way and two-way sensitivity analysis. In addition, we calculated these data for rivaroxaban and apixaban as well as for a certain range of daily costs for warfarin and daily costs of the NOACs for Germany. The cost data we used for the Markov model were comparable to those used in other countries [5, 12, 28–31, 34]. This enables a better interpretation of published findings by focusing attention on the assumptions underlying the key model features accounting for differences [39]. A real patient data analysis favoured dabigatran for stroke prophylaxis in patients with nonvalvular AF under the current hospital's perspective in a Hong Kong teaching hospital and provided a reference for further comparisons under patient and subsidization perspectives [40].

Limitations of pharmacoeconomic analyses include that they are not prespecified. Therefore the trials did not include patient-level documentation of medical resource use for estimates of total medical costs and administration of the EQ-5D for evaluation of health preferences (i.e., quality of life). The economic evaluation is more dependent on assumptions to calculate costs and the use of literature-based estimates of quality of life to generate QALYs [7]. Other limitations include differences between the studies: open [2] versus double blind study design [1, 3, 4], age, gender, creatinine clearance, CHADS2 score, history of stroke, previous therapy with warfarin, time in therapeutic range (TTR) of the INR, other biographic data of patients, and reporting minor and nonmajor bleeding complications. It has to be considered that the TTR of the INR may be higher in the studies if anticoagulation clinics such as in The Netherlands or in Italy or self-monitoring systems are used. Therefore, the individual warfarin-control groups of every study have to be used for such investigations [41]. Other limitations of the study are the extrapolation from the shorter treatment period of the studies to a 20-year-time horizon for the cost-effectiveness analysis, the fact that the willingness to pay may be set to a lower range of 20.000 to 30.000€, and the fact that in Germany no willingness-to-pay threshold exists for the health insurance system. This has to be respected for a comparison of the data across countries. The amount of willingness to pay depends substantially on the market price of the NOAC. It may be assumed that they will be reduced over time by the several economic fine-tunings. The lack of head-to-head trials makes it difficult to determine the most cost-effective agent [42]. Therefore we performed our cost-effectiveness analysis strictly only using the results of the individual studies without indirect treatment comparisons and German mortality tables to decrease the variance of the results [5].

In conclusion, edoxaban in addition to apixaban may be regarded as the most cost-effective NOAC from a German public health care insurance perspective. The larger reduction in medical cost was mainly driven by reductions in the risks major bleeding events. Additional real life use of NOAC has to substantiate the present results for specific countries, which should be collected with a support of scientific and other independent organizations.

## Figures and Tables

**Table 1:** Base-case values and ranges for probabilities of stroke, haemorrhage, myocardial infarction, and death used for sensitivity analyses of NOACs.

Variable	Edoxaban 30 mg	Edoxaban 60 mg	Reference	Dabigatran 110 mg	Dabigatran 150 mg	Reference [5]	Rivaroxaban	Reference [5]	Apixaban	Reference [5]
Stroke										
Annual rate of ischemic stroke (%)										
NOAC	1.77 (1.58–1.96)	1.25 (1.09–1.41)	[1]	1.34 (1.13–1.55)	0.92 (0.75–1.09)	[2]	1.34 (1.12–1.55)	[3]	0.97 (0.82–1.12)	[4]
Warfarin	1.25 (1.09–1.41)	1.25 (1.09–1.41)	[1]	1.2 (1.00–1.40)	1.2 (1.00–1.40)	[2]	1.42 (1.20–1.63)	[3]	1.05 (0.89–1.21)	[4]
Ischemic strokes with warfarin or NOAC (%)										
Fatal (within 30 d)	8.20 (5.50–10.90)	8.20 (5.50–10.90)	[12, 18]	8.20 (5.50–10.90)	8.20 (5.50–10.90)	[12, 18]	8.20 (5.50–10.90)	[12, 18]	8.20 (5.50–10.90)	[12, 18]
Moderate to severe neurologic sequelae	40.20 (35.30–45.10)	40.20 (35.30–45.10)	[12, 18]	40.20 (35.30–45.10)	40.20 (35.30–45.10)	[12, 18]	40.20 (35.30–45.10)	[12, 18]	40.20 (35.30–45.10)	[12, 18]
Mild neurologic sequelae	42.50 (37.60–47.40)	42.50 (37.60–47.40)	[12, 18]	42.50 (37.60–47.40)	42.50 (37.60–47.40)	[12, 18]	42.50 (37.60–47.40)	[12, 18]	42.50 (37.60–47.40)	[12, 18]
No residual neurologic sequelae	9.10 (6.20–12.00)	9.10 (6.20–12.00)	[12, 18]	9.10 (6.20–12.00)	9.10 (6.20–12.00)	[12, 18]	9.10 (6.20–12.00)	[12, 18]	9.10 (6.20–12.00)	[12, 18]
Hemorrhage										
Annual rate of ICH (%)										
NOAC	0.26 (0.18–0.34)	0.39 (0.29–0.49)	[1]	0.23 (0.14–0.32)	0.30 (0.20–0.40)	[2]	0.5 (0.37–0.63)	[3]	0.33 (0.24–0.42)	[4]
Warfarin	0.85 (0.70–0.99)	0.85 (0.70–0.99)	[1]	0.74 (0.58–0.90)	0.74 (0.58–0.90)	[2]	0.7 (0.55–0.85)	[3]	0.80 (0.66–0.94)	[4]
Annual rate of extracranial hemorrhage (%)										
NOAC	1.37 (1.19–1.55)	2.36 (2.12–2.60)	[1]	2.51 (2.23–2.79)	2.84 (2.54–3.14)	[2]	3.11 (2.78–3.44)	[3]	1.79 (1.58–2.00)	[4]
Warfarin	2.6 (2.35–2.85)	2.6 (2.35–2.85)	[1]	2.67 (2.38–2.96)	2.67 (2.38–2.96)	[2]	2.71 (2.40–3.02)	[3]	2.27 (2.03–2.51)	[4]
Annual rate of major hemorrhage (%)										
NOAC	1.61 (1.41–1.81)	2.75 (2.49–3.01)	[1]	2.71 (2.41–3.01)	3.11 (2.80–3.24)	[2]	3.6 (3.24–3.96)	[3]	2.13 (1.90–2.36)	[4]
Warfarin	3.34 (3.14–3.72)	3.34 (3.14–3.72)	[1]	3.36 (3.03–3.69)	3.36 (3.03–3.69)	[2]	3.4 (3.06–3.74)	[3]	3.09 (2.81–3.37)	[4]
Annual rate of minor hemorrhage (%)										
NOAC	6.6 (6.18–7.02)	8.67 (8.18–9.16)	[1]	13.20 (12.51–13.81)	14.80 (14.15–15.53)	[2]	11.8 (11.13–12.47)	[3]	14.03 (13.37–14.69)	[4]
Warfarin	10.15 (9.62–10.68)	10.15 (9.62–10.68)	[1]	16.40 (15.64–17.10)	16.40 (15.64–17.10)	[2]	11.4 (10.74–12.06)	[3]	19.79 (18.96–20.62)	[4]
Myocardial infarction										
Annual rate of myocardial infarction (%)										
NOAC	0.89 (0.76–1.02)	0.7 (0.58–0.82)	[1]	0.72 (0.57–0.87)	0.74 (0.59–0.89)	[2]	0.91 (0.73–1.09)	[3]	0.53 (0.42–0.64)	[4]
Warfarin	0.75 (0.63–0.87)	0.75 (0.63–0.87)	[1]	0.53 (0.40–0.66)	0.53 (0.40–0.66)	[2]	1.12 (0.92–1.32)	[3]	0.61 (0.49–0.73)	[4]
Death										
Age at start (years)	65	65	Assumption	65	65	Assumption	65	Assumption	65	Assumption
Death of cardiovascular cause (%/yr)										
NOAC	2.71 (2.48–2.94)	2.74 (2.51–2.97)	[1]	2.43 (2.15–2.71)	2.28 (2.01–2.55)	[2]	1.53 (1.30–1.76)	[3]	1.80 (1.60–2.00)	[4]
Warfarin	3.17 (2.92–3.42)	3.17 (2.92–3.42)	[1]	2.69 (2.39–2.99)	2.69 (2.39–2.99)	[2]	1.71 (1.47–1.95)	[3]	2.02 (1.81–2.23)	[4]
Death of causes other than cardiovascular or of unknown cause (%/yr)	Age adjusted from mortality tables (see reference)	Age adjusted from mortality tables (see reference)	[8]	Age adjusted from mortality tables (see reference)	Age adjusted from mortality tables (see reference)	[8]	Age adjusted from mortality tables (see reference)	[8]	Age adjusted from mortality tables (see reference)	[8]

**Table 2:** Base-case values and ranges for quality of life estimates used in sensitivity analyses for NOACs.

Variable	Edoxaban 30 mg	Edoxaban 60 mg	Reference	Dabigatran 110 mg bid	Dabigatran 150 mg bid	Reference [5]	Rivaroxaban	Reference [5]	Apixaban	Reference [5]
Quality of life estimates (utility)										
NOAC	0.994 (0.975–1.00)	0.994 (0.975–1.00)	[12, 13, 16, 18]	0.994 (0.975–1.00)	0.994 (0.975–1.00)	[12, 13, 16, 18]	0.994 (0.975–1.00)	[12, 13, 16, 18]	0.994 (0.975–1.00)	[12, 13, 16, 18]
Warfarin	0.987 (0.953–1.00)	0.987 (0.953–1.00)	[12, 16, 18]	0.987 (0.953–1.00)	0.987 (0.953–1.00)	[12, 16, 18]	0.987 (0.953–1.00)	[12, 16, 18]	0.987 (0.953–1.00)	[12, 16, 18]
Neurological sequelae										
Mild	0.87 (0.00–1.00)	0.87 (0.00–1.00)	[21]	0.87 (0.00–1.00)	0.87 (0.00–1.00)	[21]	0.87 (0.00–1.00)	[21]	0.87 (0.00–1.00)	[21]
Moderate	0.68 (0.00–1.00)	0.68 (0.00–1.00)	[21]	0.68 (0.00–1.00)	0.68 (0.00–1.00)	[21]	0.68 (0.00–1.00)	[21]	0.68 (0.00–1.00)	[21]
Serious	0.52 (0.00–1.00)	0.52 (0.00–1.00)	[21]	0.52 (0.00–1.00)	0.52 (0.00–1.00)	[21]	0.52 (0.00–1.00)	[21]	0.52 (0.00–1.00)	[21]
Recurrent event	0.12 (0.00–1.00)	0.12 (0.00–1.00)	[13]	0.12 (0.00–1.00)	0.12 (0.00–1.00)	[13]	0.12 (0.00–1.00)	[13]	0.12 (0.00–1.00)	[13]
Myocardial infarction	0.5 (0.00–1.00)	0.5 (0.00–1.00)	Assumption [12, 20]	0.5 (0.00–1.00)	0.5 (0.00–1.00)	Assumption [12, 20]	0.5 (0.00–1.00)	Assumption [12, 20]	0.5 (0.00–1.00)	Assumption [12, 20]
Hemorrhage										
Major hemorrhage	0.85 (0.00–1.00)	0.85 (0.00–1.00)	Assumption [12, 19, 22]	0.85 (0.00–1.00)	0.85 (0.00–1.00)	Assumption [12, 19, 22]	0.85 (0.00–1.00)	Assumption [12, 19, 22]	0.85 (0.00–1.00)	Assumption [12, 19, 22]
Minor hemorrhage	0.95 (0.00–1.00)	0.95 (0.00–1.00)	[12, 18]	0.95 (0.00–1.00)	0.95 (0.00–1.00)	[12, 18]	0.95 (0.00–1.00)	[12, 18]	0.95 (0.00–1.00)	[12, 18]

**Table 3:** Base-case values and ranges for costs used in sensitivity analyses for NOACs.

Variable	Edoxaban 30 mg	Edoxaban 60 mg	Ref	Dabigatran 110 mg bid	Dabigatran bid 150 mg	Ref [5]	Rivaroxaban	Ref [5]	Apixaban	Ref [5]
Costs										
Daily cost of medicine (euro)										
NOAC	3.37 (0.00–5.00)	3.37 (0.00–5.00)	Ass	3.38 (0.00–5.00)	3.38 (0.00–5.00)	[26]	3.20 (0.00–5.00)	[26]	3.54 (0.00–5.00)	[26]
Warfarin	0.20 (0.00–1.00)	0.20 (0.00–1.00)	[26]	0.20 (0.00–1.00)	0.20 (0.00–1.00)	[26]	0.20 (0.00–1.00)	[26]	0.20 (0.00–1.00)	[26]
Costs per INR determination	0.64 (0.46–0.79)	0.64 (0.46–0.79)	Ass	0.64 (0.46–0.79)	0.64 (0.46–0.79)	Ass	0.64 (0.46–0.79)	Ass	0.64 (0.46–0.79)	Ass
One-time costs of neurologic event (stroke or intracranial hemorrhage) (euro)										
Serious	7 000 (901–46 558)	7 000 (901–46 558)	[14]	7 000 (901–46 558)	7 000 (901–46 558)	[14]	7 000 (901–46 558)	[14]	7 000 (901–46 558)	[14]
Moderate	4 233 (901–46 558)	4 233 (901–46 558)	[14]	4 233 (901–46 558)	4 233 (901–46 558)	[14]	4 233 (901–46 558)	[14]	4 233 (901–46 558)	[14]
Mild	3 942 (2 014–4 233)	3 942 (2 014–4 233)	[14]	3 942 (2 014–4 233)	3 942 (2 014–4 233)	[14]	3 942 (2 014–4 233)	[14]	3 942 (2 014–4 233)	[14]
One-time costs for myocardial infarction (euro)	10 000 (2 743–48 023)	10 000 (2 743–48 023)	[14]	10 000 (2 743–48 023)	10 000 (2 743–48 023)	[14]	10 000 (2 743–48 023)	[14]	10 000 (2 743–48 023)	[14]
One-time costs for hemorrhage (euro)										
Major hemorrhage	2 500 (891–5 415)	2 500 (891–5 415)	[23]	2 500 (891–5 415)	2 500 (891–5 415)	[23]	2 500 (891–5 415)	[23]	2 500 (891–5 415)	[23]
Minor hemorrhage	50 (0.00–100)	50 (0.00–100)	Ass	50 (0.00–100)	50 (0.00–100)	Ass	50 (0.00–100)	Ass	50 (0.00–100)	Ass
Rehabilitation costs (euro)										
Annual ambulant rehabilitation costs	2 300 (1 800–2 800)	2 300 (1 800–2 800)	[43]	2 300 (1 800–2 800)	2 300 (1 800–2 800)	[43]	2 300 (1 800–2 800)	[43]	2 300 (1 800–2 800)	[43]
Inpatient rehabilitation costs per patient	8 000 (2 000–14 000)	8 000 (2 000–14 000)	[43]	8 000 (2 000–14 000)	8 000 (2 000–14 000)	[43]	8 000 (2 000–14 000)	[43]	8 000 (2 000–14 000)	[43]
Annual costs for further medical treatment	2 900 (2 300–4 000)	2 900 (2 300–4 000)	[43]	2 900 (2 300–4 000)	2 900 (2 300–4 000)	[43]	2 900 (2 300–4 000)	[43]	2 900 (2 300–4 000)	[43]
Costs in case of death (euro)	2 500	2 500	[14]	2 500	2 500	[14]	2 500	[14]	2 500	[14]
Discounting (%)	5 (0–10)	5 (0–10)	[9, 10, 24]	5 (0–10)	5 (0–10)	[9, 10, 24]	5 (0–10)	[9, 10, 24]	5 (0–10)	[9, 10, 24]

Ref = reference; Ass = assumption.

**Table 4:** Results of the base-case analysis for a 65-year-old population over a time horizon of 20 years from a German healthcare insurance perspective.

Trial	Anticoagulant	QALY	Total costs *€*	ICER *€*/QALY	Daily price *€*/d	PSA ICER *€*/QALY
ENGAGE-AF	Edoxaban 30 mg od	7.65	21 052	68 275	3.37	69 600
	Warfarin	7.48	9 747		0.20	
	Edoxaban 60 mg od	7.69	20 157	50 411	3.37	52 000
	Warfarin	7.48	9 747		0.20	

RE-LY [5]	Dabigatran 110 mg bid	7.68	20 048	294 349	3.38	278 000
	Warfarin	7.64	7 622		0.20	
	Dabigatran 150 mg bid	7.71	19 537	163 184	3.38	174 000
	Warfarin	7.64	7 622		0.20	

ROCKET-AF [5]	Rivaroxaban 20 mg od	7.67	19 874	133 926	3.20	130 500
	Warfarin	7.59	9 069		0.20	

ARISTOTLE [5]	Apixaban 5 mg bid	7.75	19 885	57 245	3.54	55 500
	Warfarin	7.56	8 915		0.20	

## References

[1] Giugliano R. P., Ruff C. T., Braunwald E. (2013). Edoxaban versus warfarin in patients with atrial fibrillation. *The New England Journal of Medicine*.

[2] Connolly S. J., Ezekowitz M. D., Yusuf S. (2009). Dabigatran versus warfarin in patients with atrial fibrillation. *The New England Journal of Medicine*.

[3] Patel M. R., Mahaffey K. W., Garg J. (2011). Rivaroxaban versus warfarin in nonvalvular atrial fibrillation. *The New England Journal of Medicine*.

[4] Granger C. B., Alexander J. H., McMurray J. J. (2011). Apixaban versus warfarin in patients with atrial fibrillation. *The New England Journal of Medicine*.

[5] Krejczy M., Harenberg J., Marx S., Obermann K., Frölich L., Wehling M. (2014). Comparison of cost-effectiveness of anticoagulation with dabigatran, rivaroxaban and apixaban in patients with non-valvular atrial fibrillation across countries. *Journal of Thrombosis and Thrombolysis*.

[6] Drummond M., Barbieri M., Cook J. (2009). Transferability of economic evaluations across jurisdictions: ISPOR Good Research Practices Task Force report. *Value in Health*.

[7] Reed S. D. (2013). How country-specific should a country-specific cost-effectiveness analysis be?. *European Heart Journal*.

[8] Eikelboom J. W., Hirsh J., Spencer F. A., Baglin T. P., Weitz J. I. (2012). Antiplatelet drugs: antithrombotic therapy and prevention of thrombosis, 9th ed: American College of Chest Physicians evidence-based clinical practice guidelines. *Chest*.

[9] Weinstein M. C., Siegel J. E., Gold M. R., Kamlet M. S., Russell L. B. (1996). Recommendations of the panel on cost-effectiveness in health and medicine. *The Journal of the American Medical Association*.

[10] Adam H., Ahlert M., Breyer F. (2009). *Dokumentation der Stellungnahmen zum “Entwurf einer Methodik für die Bewertung von Verhältnissen zwischen Nutzen und Kosten im System der deutschen gesetzlichen Krankenversicherung Version 2.0”*.

[11] Lip G. Y. H., Rasmussen L. H., Olsson S. B. (2009). Oral direct thrombin inhibitor AZD0837 for the prevention of stroke and systemic embolism in patients with non-valvular atrial fibrillation: a randomized dose-guiding, safety, and tolerability study of four doses of AZD0837 vs. vitamin K antagonists. *European Heart Journal*.

[12] Freeman J. V., Zhu R. P., Owens D. K. (2011). Cost-effectiveness of dabigatran compared with warfarin for stroke prevention in atrial fibrillation. *Annals of Internal Medicine*.

[13] Shah S. V., Gage B. F. (2011). Cost-effectiveness of dabigatran for stroke prophylaxis in atrial fibrillation. *Circulation*.

[14] http://www.g-drg.de/cms/G-DRG-System_2011/Fallpauschalen-Katalog/Fallpauschalen-Katalog_2011;abgerufenam10.07.2011.

[15] Drummond M. F., Sculpher M. J., Torrance G. W., O'Brien B. J., Stoddart G. L. (2005). *Methods for the Economic Evaluation of Health Care Programmes*.

[16] Gage B. F., Cardinalli A. B., Owens D. K. (1996). The effect of stroke and stroke prophylaxis with aspirin or warfarin on quality of life. *Archives of Internal Medicine*.

[17] Hay J. W., Smeeding J., Carroll N. V. (2010). Good research practices for measuring drug costs in cost effectiveness analyses: Issues and recommendations: the ISPOR drug cost task force report. Part I. *Value in Health*.

[18] O'Brien C. L., Gage B. F. (2005). Costs and effectiveness of ximelagatran for stroke prophylaxis in chronic atrial fibrillation. *The Journal of the American Medical Association*.

[19] Fryback D. G., Dasbach E. J., Klein R. (1993). The Beaver Dam Health Outcomes Study: initial catalog of health-state quality factors. *Medical Decision Making*.

[20] Sullivan P. W., Ghushchyan V. (2006). Preference-based EQ-5D index scores for chronic conditions in the United States. *Medical Decision Making*.

[21] Tengs T. O., Lin T. H. (2003). A meta-analysis of quality-of-life estimates for stroke. *PharmacoEconomics*.

[22] Thomson R., Parkin D., Eccles M., Sudlow M., Robinson A. (2000). Decision analysis and guidelines for anticoagulant therapy to prevent stroke in patients with atrial fibrillation. *The Lancet*.

[23] Bufe A., Frey S., Briswalter S. (2009). Durch Blutungen verursachte Kosten bei der Therapie des akuten Koronarsyndroms in Deutschland. *Herz Kardiovaskuläre Erkrankungen*.

[24] Canadian Agency for Drugs and Technologies in Health (2006). *Guidelines for the Economic Evaluation of Health Technologies: Canada*.

[25] Graf von der Schulenburg J. M., Greiner W., Jost F. (2007). German recommendations on health economic evaluation—third and updated version of the Hanover Consensus. *Gesundheitsokonomie und Qualitatsmanagement*.

[26] van Ryn J., Stangier J., Haertter S. (2010). Dabigatran etexilate—a novel, reversible, oral direct thrombin inhibitor: interpretation of coagulation assays and reversal of anticoagulant activity. *Thrombosis and Haemostasis*.

[27] Hein L. (2011). Antithrombotika und Antihämorrhagika. *Arzneimittelverordnungs-Report 2011*.

[28] Sorensen S. V., Kansal A. R., Connolly S. (2011). Cost-effectiveness of dabigatran etexilate for the prevention of stroke and systemic embolism in atrial fibrillation: a Canadian payer perspective. *Thrombosis and Haemostasis*.

[29] Pink J., Lane S., Pirmohamed M., Hughes D. A. (2011). Dabigatran etexilate versus warfarin in management of non-valvular atrial fibrillation in UK context: quantitative benefit-harm and economic analyses. *The Britich Medical Journal*.

[30] Kansal A. R., Sorensen S. V., Gani R. (2012). Cost-effectiveness of dabigatran etexilate for the prevention of stroke and systemic embolism in UK patients with atrial fibrillation. *Heart*.

[31] Langkilde L. K., Bergholdt Asmussen M., Overgaard M. (2012). Cost-effectiveness of dabigatran etexilate for stroke prevention in non-valvular atrial fibrillation. Applying RE-LY to clinical practice in Denmark. *Journal of Medical Economics*.

[32] Davidson T., Husberg M., Janzon M., Oldgren J., Levin L. A. (2013). Cost-effectiveness of dabigatran compared with warfarin for patients with atrial fibrillation in Sweden. *European Heart Journal*.

[33] Silva Miguel L., Rocha E., Ferreira J. (2013). Economic evaluation of dabigatran for stroke prevention in patients with non-valvular atrial fibrillation. *Revista Portuguesa de Cardiologia*.

[34] Lee S., Anglade M. W., Pham D., Pisacane R., Kluger J., Coleman C. I. (2012). Cost-effectiveness of *Rivaroxaban* compared to *Warfarin* for stroke prevention in atrial fibrillation. *American Journal of Cardiology*.

[35] Harrington A. R., Armstrong E. P., Nolan P. E., Malone D. C. (2013). Cost-effectiveness of apixaban, dabigatran, rivaroxaban, and warfarin for stroke prevention in atrial fibrillation. *Stroke*.

[36] Coyle D., Coyle K., Cameron C. (2013). Cost-effectiveness of new oral anticoagulants compared with warfarin in preventing stroke and other cardiovascular events in patients with atrial fibrillation. *Value in Health*.

[37] Rognoni C., Marchetti M., Quaglini S., Liberato N. L. (2014). Apixaban, dabigatran, and rivaroxaban versus warfarin for stroke prevention in non-valvular atrial fibrillation: a cost-effectiveness analysis. *Clinical Drug Investigation*.

[38] Kansal A. R., Sharma M., Bradley-Kennedy C. (2012). Dabigatran versus rivaroxaban for the prevention of stroke and systemic embolism in atrial fibrillation in Canada. Comparative efficacy and cost-effectiveness. *Thrombosis and Haemostasis*.

[39] Sorensen S. V., Peng S., Monz B. U., Bradley-Kennedy C., Kansal A. R. (2013). A comparative analysis of models used to evaluate the cost-effectiveness of dabigatran versus warfarin for the prevention of stroke in atrial fibrillation. *PharmacoEconomics*.

[40] Chang A. M., Ho J. C. S., Yan B. P., Yu C. M., Lam Y. Y., Lee V. W. Y. (2013). Cost-effectiveness of dabigatran compared with warfarin for stroke prevention in patients with atrial fibrillation—a real patient data analysis in a Hong Kong teaching hospital. *Clinical Cardiology*.

[41] Harenberg J., Marx S., Diener H.-C. (2012). Comparison of efficacy and safety of dabigatran, rivaroxaban and apixaban in patients with atrial fibrillation using network meta-analysis. *International Angiology*.

[42] Limone B. L., Baker W. L., Kluger J., Coleman C. I. (2013). Novel anticoagulants for stroke prevention in atrial fibrillation: a systematic review of cost-effectiveness models. *PLoS ONE*.

[43] Claes C., Mittendorf T., Grond M., Graf von der Schulenburg J.-M. (2009). Inkrementelle Kosteneffektivität von Dipyridamol + Acetylsalicylsäure in der Sekundärprävention bei ischämischem nichtkardioembolischem Schlaganfall. *Medizinische Klinik*.

